# A comparison of artificial intelligence techniques for predicting hyperforin content in *Hypericum perforatum* L. in different ecological habitats

**DOI:** 10.1002/pld3.363

**Published:** 2021-11-24

**Authors:** Maryam Saffariha, Ali Jahani, Reza Jahani

**Affiliations:** ^1^ College of Natural Resources University of Tehran Tehran Iran; ^2^ Assessment and Environment Risks Department Research Center of Environment and Sustainable Development Tehran Iran; ^3^ Department of Pharmacology and Toxicology, School of Pharmacy Shahid Beheshti University of Medical Sciences Tehran Iran

**Keywords:** artificial intelligence, ecological modeling, graphical user interface, hyperforin, *Hypericum perforatum*

## Abstract

Hyperforin, a major bioactive constituent of *Hypericum* concentration, is impacted by various phenological phases and soil characteristics. We aimed to design a model predicting hyperforin content in *Hypericum perforatum* based on different ecological and phenological conditions. We employed artificial intelligence modeling techniques including multilayer perceptron (MLP), radial basis function (RBF), and support vector machine (SVM) to examine the factors critical in predicting hyperforin content. We found that the MLP model (*R* ^2^ = .9) is the most suitable and precise model compared with RBF (*R* ^2^ = .81) and SVM (*R* ^2^ = .74) in predicting hyperforin in *H. perforatum* based on ecological conditions, plant growth, and soil features. Moreover, phenological stages, organic carbon, altitude, and total N are detected in sensitivity analysis as the main factors that have a considerable impact on hyperforin content. We also report that the developed graphical user interface would be adaptable for key stakeholders including producers, manufacturers, analytical laboratory managers, and pharmacognosists.

## 1 INTRODUCTION

*Hypericum perforatum* L., commonly known as St John's wort, belongs to the family of Hypericaceae. This family has been studied widely as a source of bioactive compounds utilized as complementary therapeutics for weak to mild depression (Barnes et al., 2019). In addition, numerous preclinical and clinical studies have demonstrated anxiolytic, sedative, nootropic, antischizophrenic, anticonvulsant, anti‐inflammatory, antibacterial, and antiviral activities (Galeotti, 2017). *H. perforatum* is one of the most important medicinal plant species of the family Hypericaceae and is listed as a top‐selling herbal preparation due to presence of hyperforin, hypericin, and pseudohypericin along with other biologically active metabolites (Christenhusz & Byng, 2016). Hyperforin is a prenylated phloroglucinol derivative that contains a phloroglucinol skeleton with lipophilic isoprene chains (Ramalhete et al., 2016). For many years, it was believed that hypericin is responsible for the antidepressant properties of *H. perforatum*. However, recent clinical trials have demonstrated that hyperforin also contributes to alleviate depression symptoms (Szewczyk et al., 2019). Recently, hyperforin is studied to exhibit various other pharmacological benefits such as anticancer (Zhao et al., 2015), neuroprotective (Gaid et al., 2018), anti‐inflammatory (Khan et al., 2019), antiangiogenic (Nabavi et al., 2018), and antibacterial properties. Therefore, accurate identification and subsequent quantification of hyperforin is imperative for testing these activities and eventually developing a commercial preparation (Barnes et al., 2019). High‐performance liquid chromatography (HPLC) with different detectors such as diode array, fluorescence detector, and mass spectrometry has been employed traditionally for routine detection and quantifications of hyperforin (Gitea et al., 2018). These instrumentations are accurate for identification of secondary metabolites (Saffariha et al., 2020), but they also present some challenges. For instance, complex data analysis procedures require time and trained human resource and consumption of high volume of reagents and solvents (Sahu et al., 2018). Thus, there is a dearth of relatively inexpensive and robust method and/or tools for accurate prediction of hyperforin content in *H. perforatum* applicable to various ecological habitats.

*H. perforatum* can adapt to a range of environmental conditions by changing its metabolic profile (Murch & Saxena, 2006). Various factors including soil composition, genetic, environmental conditions, and phenological stages are reported to impact the content of bioactive compounds in medicinal plants (Cirak & Radusiene, 2019; Radušienė et al., 2012). Similarly, content of hypericin, pseudohypericin, and hyperforin in *Hypericum perfoliatum* L., *Hypericum montbretii* Spach, and *Hypericum origanifolium* Willd is studied to be impacted significantly by different ecological conditions (Cirak et al., 2007; Cirak et al., 2008; Cirak & Radusiene, 2007; Maggi & Cecchini, 2010). We reported that the highest essential oil yield of *Salvia limbata* L. at flowering stage at the highest altitude (Saffariha et al., 2019). Radušienė et al. (2012) recorded the highest levels of hyperforin at flowering stage using liquid chromatography–mass spectrometry (LC–MS). Alternatively, artificial intelligence techniques are promising in accurately predicting content of secondary metabolites in medicinal plants (Saffariha et al., 2021).

Artificial neural network (ANN) modeling techniques, also known as artificial intelligence techniques, have been designed based on human brain functions using various mathematical algorithms to obtain maximum accuracy in outputs. Support vector machine (SVM), multilayer perceptron (MLP) neural network, and radial basis function (RBF) are the three most frequently used techniques of artificial intelligence in the field of chemical ecology (Kalantary et al., 2020). Recently, these nonlinear techniques have been compared to achieve the most accurate model in the prediction of the ecological process (Aghajani et al., 2014; Jahani & Saffariha, 2020b). For instance, the seed germination percentage of *S*. *limbata* was predicted under different ecological stresses (Saffariha et al., 2020) by comparing ANN models. ANN‐based models can also potentially assist researchers to predict hyperforin content in *H. perforatum* with relatively less resources. These models also need to be validated across various ecological conditions to enhance their applicability. Saraiva et al. (2018) determined the effects of CO_2_ enrichment on the growth and biometal/nutrient content and accumulation in *Senna reticulate*. An ANN accurately predicted results suggesting that Mg, Na, and Fe contents display the most different behavior when comparing plants germinated at atmospheric and elevated CO_2_ conditions. Also, Saffariha et al. (2021) measured hypericin content in *H. perforatum* and tested the potential of artificial intelligence techniques to correlate ecological factors with hypericin content. They found the MLP model (*R* ^2^ = .87) as the most accurate ANN technique in hypericin content prediction, but they believe that application of MLP technique for valid prediction of plants biochemical contents needs to be more explored in other biochemical contents to ensure the results. Rajkovic et al. (2013) used ANN techniques to optimize models for prediction of the sunflower oil transesterification. Authors compared the performances of the models as a decision support system tool during the investigated methanolysis process. The fatty acid methyl ester yield was predicted by ANN model much better than the predictions (±24.2%) obtained by the second‐order polynomial equation.

Therefore, the aim of this research was to compare MLP, RBF, and SVM to predict the amount of hyperforin in *H. perforatum* at different growth stages and ecological conditions. The best model among the proposed models determines the most important ecological and phenological factors in the amount of hyperforin in *H. perforatum*. Also using the graphical user interface (GUI) tool will be able researchers to define the amount of hyperforin in *H. perforatum*. Moreover, our findings promoted a commercial consumption of active ingredients in *H. perforatum*.

## 2 MATERIALS AND METHODS

### 2.1 Study area and sampling

The study area is located in the south of Alborz Mountain in Alborz Province in the north of Iran (35° 44′N to 36° 35′N and 51° 00′E to 51° 36′E). This area is considered as most suitable habitats of *H. perforatum* in the range of 1000–4000 m altitude. To include considerable variability in ecological conditions, 100 aerial tissue samples were collected along 15 transects of 1000 m at different altitudes at three phenological stages (vegetative growth, flowering, and seeding) over the period of 6 months. Some soil properties such as electrical conductivity (EC), absorbable phosphor (ppm), acidity (pH), sand (%), silt (%), clay (%), absorbable potassium (ppm), organic carbon (%), and total nitrogen (%) were recorded at each sample location. Other features such as altitude (m), slope (%), and hill aspect (four aspects including 1. North, 2. East, 3. South, and 4. West) for each sample were recorded. Finally, samples were sent to the Herbarium of Medicinal Plants and Drugs Research Institute, Shahid Beheshti University, Tehran, Iran, for further analysis.

### 2.2 Chemical and reagents

**FIGURE 1:**
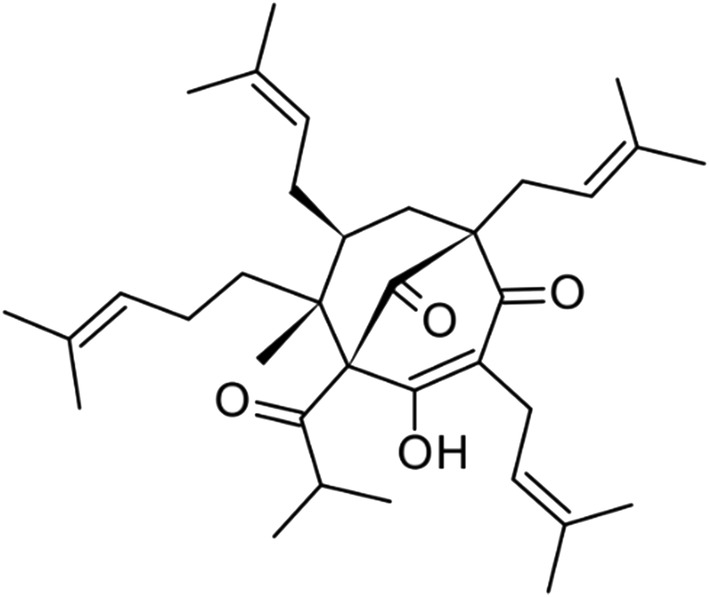
Chemical structures of the hyperforin

Hyperforin (Figure 1, 99% purity) was purchased from Alexis Corp. (Lausen, Switzerland). (2‐Hydroxypropyl)‐*β*‐cyclodextrin and ortho‐phosphoric acid (H_3_PO_4_) were obtained from Sigma (St. Louis, MO, USA). HPLC grade methanol and acetonitrile were purchased from Merck (Darmstadt, Germany), and pure water was obtained from a Milli‐Q water purification system (Millipore, Bedford, MA, USA).

### 2.3 Extraction of *H. perforatum*

Hyperforin extraction was performed using the method described by Soelberg et al. (2007). Briefly, fresh plant leaves were frozen with liquid nitrogen and ground into a very fine powder. Then, 250 mg of the prepared leaf powder was added to 2.5 ml of 80% aqueous methanol containing 0.073 M (2‐hydroxypropyl)‐*β*‐cyclodextrin in a centrifuge tube, and the pH was adjusted to 2.5 with H_3_PO_4_. The centrifuge tubes were sonicated for 10 min in an ultrasonic bath (Elma S30H, Germany) before 10‐min centrifugation at 5000 rpm. The supernatant was decanted into a 10‐ml volumetric flask. This procedure was repeated two times with the pellet, and the obtained supernatants were mixed. Finally, the volume of the collected supernatant was adjusted to the constant volume of 10 ml with methanol, and the sample was filtered through a .45‐μm PFTE filter (Gelman Sciences, Ann Arbor, MI, USA).

### 2.4 HPLC analysis of hyperforin

An Agilent Series 1200 HPLC system (Agilent Corporation, Palo Alto, CA, USA) that was equipped with a G1312A bin pump, a G1379B online degasser, a Mightysil RP‐18 GP column (5 μm, 250 × 4.6 mm, Kanto Chemical, Tokyo, Japan) and a G1314B ultraviolet detector (Agilent Corporation, Palo Alto, CA, USA) was used for the analysis of hyperforin in the collected *H. perforatum* samples. For this purpose, a mixture containing acetonitrile and 0.3% phosphoric acid in water at a ratio of 90:10 was used as the mobile phase. Injection volume, flow rate, column oven temperature, and detector wavelength were set at 20 μl, 1.5 ml/min, 30°C, and 273 nm, respectively (Kuo et al., 2020). The hyperforin content was calculated using the plotted standard calibration curve (concentration vs. peak area) and expressed as mg/g dry mass following triplicate measurement.

### 2.5 Modeling process

The current study aimed to study variations in the amount of hyperforin arising due to different landscapes, soils, and phenological stages in Alborz protected area, and current methods are incapable of providing high‐precision data. According to studies, ANN models provide more accurate predictions in ecological phenomena studies (Jahani & Rayegani, 2020). Thus, the ANN function in MATLAB 2018 was used to design the structure of three models (MLP, RBF, and SVM).

### 2.6 Multilayer perceptron neural network

Neurons are the main elements used in the MLP model (Shams et al., 2020, 2021). To get an accurate model, hidden layers, transfer functions, and neurons must be carefully analyzed. In this paper, input variables include landform, phenological stages, and soil characteristics, and the output variable is the content of hyperforin. The MLP model by weighting variables and summarizing them produced the most accurate output in previous study by Shams et al. (2020, 2021) and Pourmohammad et al. (2020). At first, 60% of samples put in use in the training process. The remaining 40% of samples were divided equally in two data sets (20, 20) for validation and testing (Mosaffaei & Jahani, 2020). Most of studies apply 60–70% of samples for training, and the rest of them are tagged as validation and test data (Cline et al., 2018; Jahani & Rayegani, 2020; Kalantary et al., 2020; Saraiva et al., 2018). On the other hand, the ANN specialist and developer believe that the volume of validation data should be half of training samples (refer to Demuth & Beale, 2002). In MLP training, the weights (*w*) of the *i*th variable (*x*) in *j*th neuron are defined to calculate the output of *j*th neuron on the *k*th hidden layer ( ${\mathrm{net}}_{j}^{k}$) by Equation 1.

(1) $${\mathrm{net}}_{j}^{k}=\sum_{i=0}^{n}w_{\mathrm{ji}}x_{\mathrm{ji}}$$

We considered the output of Equation 1 as the input of a transfer function (*∫*) in Equation 2. Many different functions are tested for the most accurate output (Demuth & Beale, 2002).

(2) $$Y_{\mathrm{net}}=\int {\mathrm{net}}_{j}$$

The accurate weighing of neurons and layers was carefully evaluated, and the most appropriate weights were selected. To accurately calculate hyperforin content in *H. perforatum* samples, we applied the back propagation method in Equation 3. In Equation 3, *E* presents the sum of squared errors, *w*_*ji*_ illustrates the weight of *i*th neuron in *j*th hidden layer, and *ᵧ* is the learning rate which is determined by a crisp value (refer to Demuth & Beale, 2002).

(3) $$w_{\mathrm{ji}}^{t}=w_{\mathrm{ji}}^{t-1}+\left(-ᵧ\frac{\partial E^{t}}{\partial w_{\mathrm{ji}}^{t}}\right)$$

### 2.7 RBF neural network

Although a set of neurons was used in RBF, in general, RBF acts as a transfer function. In the modeling process, 80% of all samples were used to train the network, and the remaining 20% were allocated for the accuracy assessment of the network. One of the most applicable RBF functions is Gaussian (Kalantary et al., 2020), and we used this function in our model. The center of circular classifiers, in multidimensional space, is measured by Equation 4.

(4) $$R_{j}\left(x\right)=\exp\left(\frac{║x-a_{j}{║}^{2}}{2\sigma^{2}}\right)$$

In Equation 4, *R*_j_(*x*) = the RBF, ||*x*_*a*_*j*_|| = the determined Euclidean distance between the total of *a*_*j*_ (RBF function center), *x* = (input vector or variables), and *σ* = a positive real number.

Finally, to predict hyperforin content, we employed Equation 5.

(5) $$y_{k}=\sum_{j=1}^{m}w_{\mathrm{jk}}R_{j}\left(x\right)+b_{j}$$

In Equation 5, *w*_*ik*_ = the weights of neurons, *j* = the number of each node in the hidden layer, *m* = the number of neurons, and *b*_*j*_ = bias (Kalantary et al., 2020).

### 2.8 Support vector machine

SVM is known as a classifier with a set of margins for determining the boundaries of classification which cover the uncertainties in the outputs of the model. In the modeling process, our ultimate goal was to optimize classification boundaries. As we explained in the RBF model, 80% of all samples were allocated to train the network, and the remaining samples were used for the accuracy assessment of the network. The SVM model (Equation 6) uses input variables in the structure of a kernel function (Equation 7).

(6) $$y\left(x\right)=\sum_{i=1}^{n}\alpha_{i}K\left(x_{i},x_{j}\right)+b$$

There is a kernel function that is explained in Equation 7. The parameters of Equation 7 are *x*_*i*_ and *x*_*j*_ = samples and *γ* = kernel parameter.

(7) $$K\left(x_{i},x_{j}\right)=\exp\left(-\gamma {\left\|x_{i}-x_{j}\right\|}^{2}\right)$$

The kernel function parameters are *x*_*i*_ and *x*_*j*_ = samples and *γ* = kernel parameter. By minimizing SVM network errors, we balanced the weight of the network to predict outputs (Equation 8). In Equation 8, the parameters were Σξ_*i*_ = training errors, 1/2||*w*||2 = the margin, and *C* = the tuning parameter.

(8) $$\frac{1}{2}{\left\|w\right\|}^{2}+C\sum_{i=1}^{n}\xi_{i}$$

### 2.9 Accuracy assessment of models

We employed the test data set to analyze the performance of the models. Based on recent research, statistical indicators have been used to assess the accuracy of the model (e.g., Jahani & Saffariha, 2020b). These indicators are MSE (Equation 9), RMSE (Equation 10), MAE (Equation 11), and *R* ^2^ (Equation 12). In these equations, $y_{i}$ and ${\hat{y}}_{i}$ = the targets and network outputs, respectively, ${\bar{y}}_{i}$ = the mean of target values, and *N* = the number of samples.

(9) $$\mathrm{MSE}=\frac{\sum_{i=1}^{n}{\left(y_{i}-{\hat{y}}_{i}\right)}^{2}}{n}$$

(10) $$\text{RMSE}=\sqrt{\frac{\sum_{i=1}^{n}{\left(y_{i}-{\hat{y}}_{i}\right)}^{2}}{n}}$$

(11) $$\mathrm{MAE}=\frac{\sum_{i=1}^{n}\left|y_{i}-{\hat{y}}_{i}\right|}{n}$$

(12) $$R^{2}=\frac{\sum_{i=1}^{n}{\left({\hat{y}}_{i}-{\bar{y}}_{i}\right)}^{2}}{\sum_{i=1}^{n}{\left(y_{i}-{\bar{y}}_{i}\right)}^{2}}$$

### 2.10 Sensitivity analysis

Different values of input variables in three developed models have a linear or logarithmic effect on the output. To prioritize the variables affecting the content of hyperforin in *H. perforatum*, we conducted a sensitivity analysis. In this method, each variable has its data set, and the range of standard deviation was variable. We changed each variable in the specified range, and other variables were fixed (equal to average). The sensitivity value for each variable was calculated. This value is equal to the standard deviation of outputs in response to variable changes.

### 2.11 GUI tool

A GUI as an EDSS tool was designed to determine the most precise model to predict the content of hyperforin in *H. perforatum*. In this tool, by applying ecological conditions of the site, landform, soil characteristics, and plant phenological stages, the content of hyperforin in *H. perforatum* was predicted.

## 3 RESULTS

### 3.1 MLP performance

We used three datasets to obtain the best results from the MLP model. To predict the most precise MLP model, the number of neurons, hidden layers, training method, and activation function has been modified. Table 1 illustrates that the structure 13‐8‐1 has been considered as the most authentic in the prediction of hyperforin content in *H. perforatum*. The factors used for the MLP model include 13 inputs, 8 neurons in the hidden layer, and 1 neuron as output.

**TABLE 1:** The results of parameters tuning in multilayer perceptron (MLP) structure

Activation function	Training function	Structure	Test set				Training data			
			*R* ^2^	MSE	RMSE	MAE	*R* ^2^	MSE	RMSE	MAE
Logsig‐Purelin	LM	13‐8‐1	.90	0.14	0.37	0.32	.91	0.10	0.01	0.26

**FIGURE 2:**
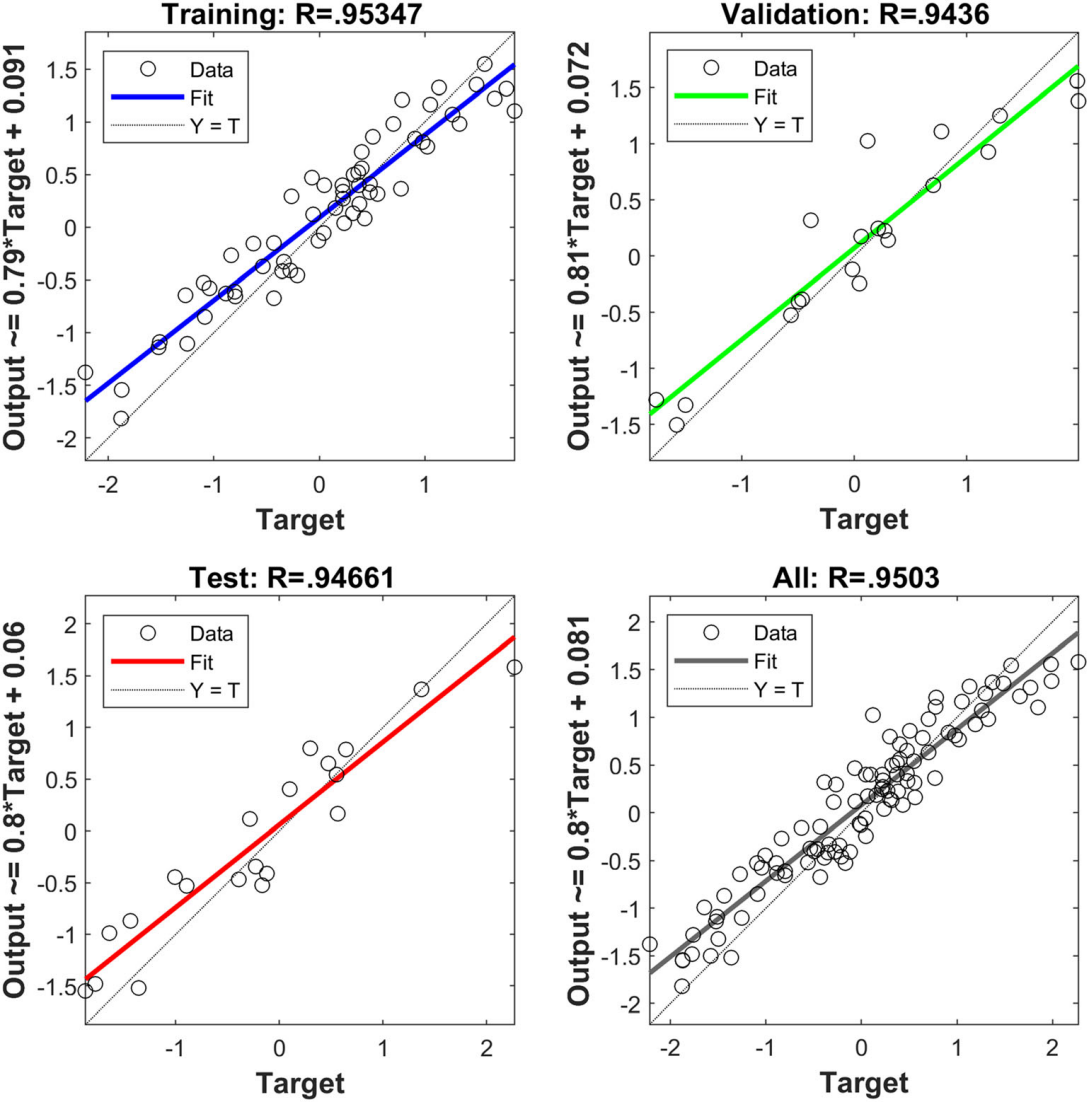
Scatter plots of targets values of hyperforin content generated through multilayer perceptron (MLP) outputs

We applied the scatter plot to create a correlation between the target and MLP output (Jahani & Saffariha, 2020b). In Figure 2, the scatter plot of MLP outputs versus targets values of the hyperforin content for training, validation, test, and total data is shown. According to coefficient (*R* ^2^), there is a considerable correlation between MLP outputs and target values.

**FIGURE 3:**
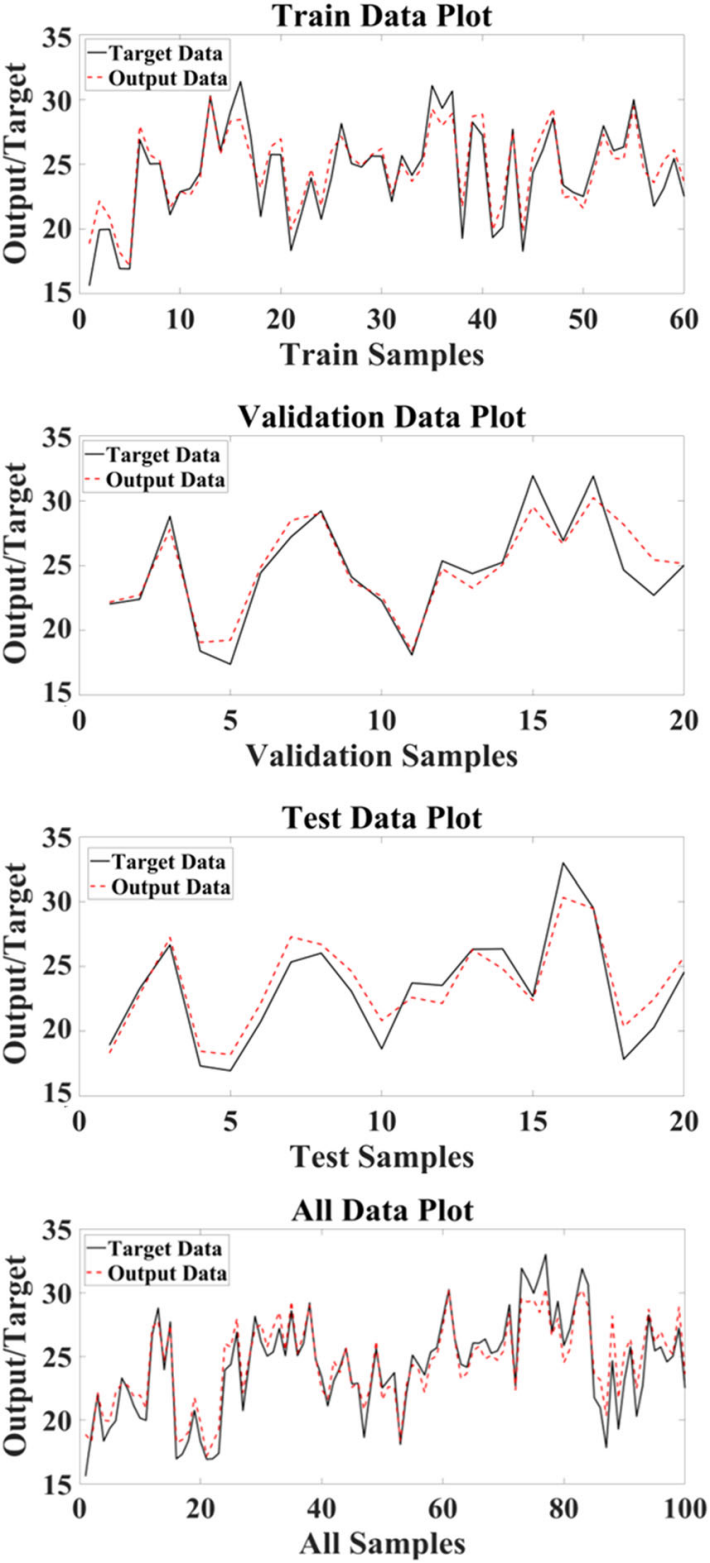
The target hyperforin content and the outputs of multilayer perceptron (MLP) in datasets

There is a comparison between the real (target) and simulated (output) values of MLP in the datasets shown in Figure 3.

### 3.2 RBF performance

In the process of training data, there are factors such as spread and neurons that needed to be optimized. In the training phase, we seek to reduce network error through spread values. Therefore, the number of neurons was 13, and the spread of RBF was 6.4 to attain the best performance. In Table 2, the results of RBF in training and test datasets are displayed. The best structure for the value of *R* ^2^ in training and test datasets is listed in Table 2. The structure of optimized RBF is 13‐13‐1 with 13 variables as inputs, 13 neurons in the hidden layer with Gaussian transfer function, and 1 neuron of output.

**TABLE 2:** The RBF structure results (spread and neurons) in training and test data sets

Model	Spread	Neurons	Test data				Training data			
			*R* ^2^	MSE	RMSE	MAE	*R* ^2^	MSE	RMSE	MAE
RBF	6.4	13	.81	0.15	0.39	0.34	.88	0.12	0.35	0.29

Abbreviation: RBF, radial basis function.

**FIGURE 4:**
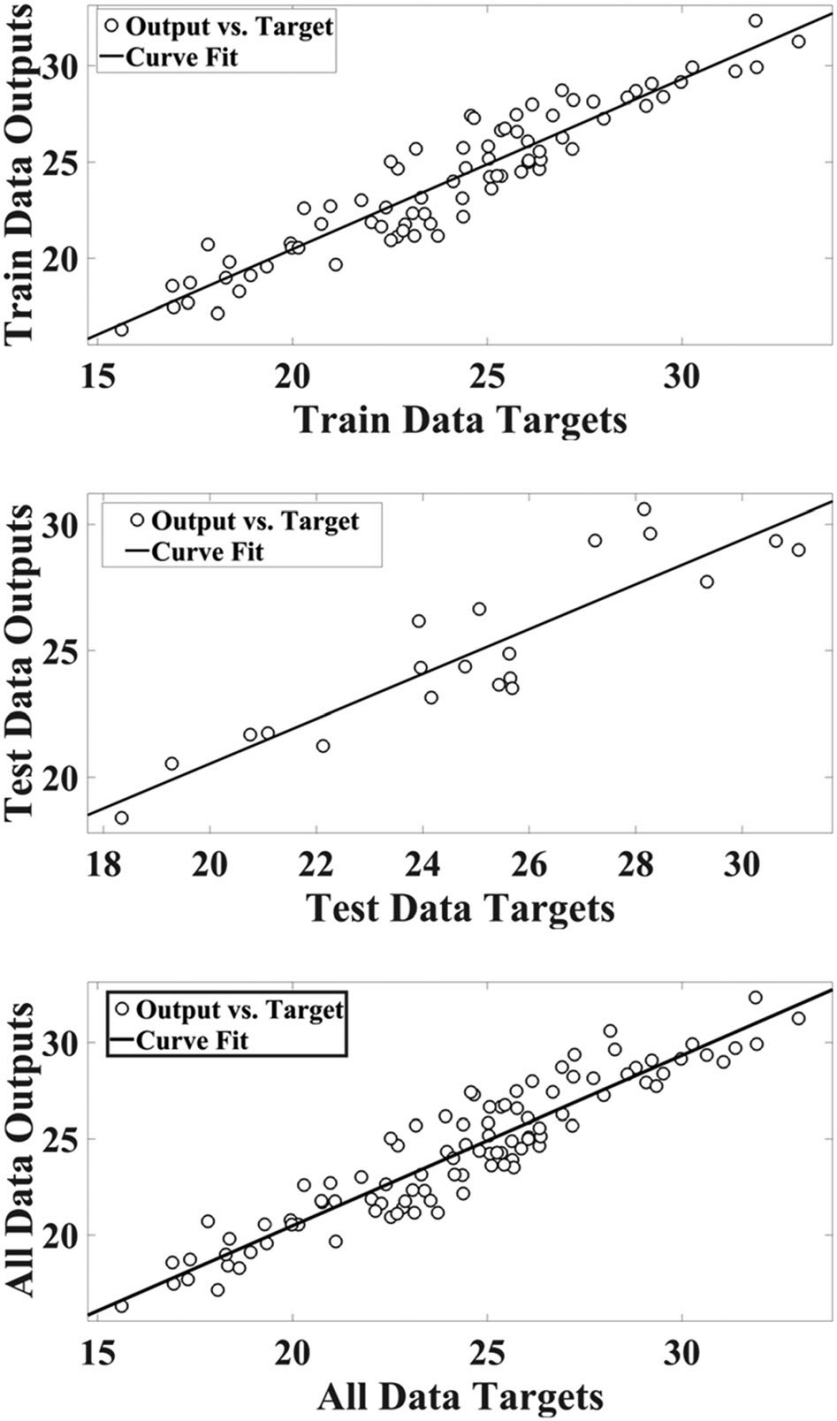
Scatter plots of targets values of hyperforin content via radial basis function (RBF) outputs

A scatter plot of RBF outputs versus targets values of hyperforin content for training, test, and total data is shown in Figure 4.

**FIGURE 5:**
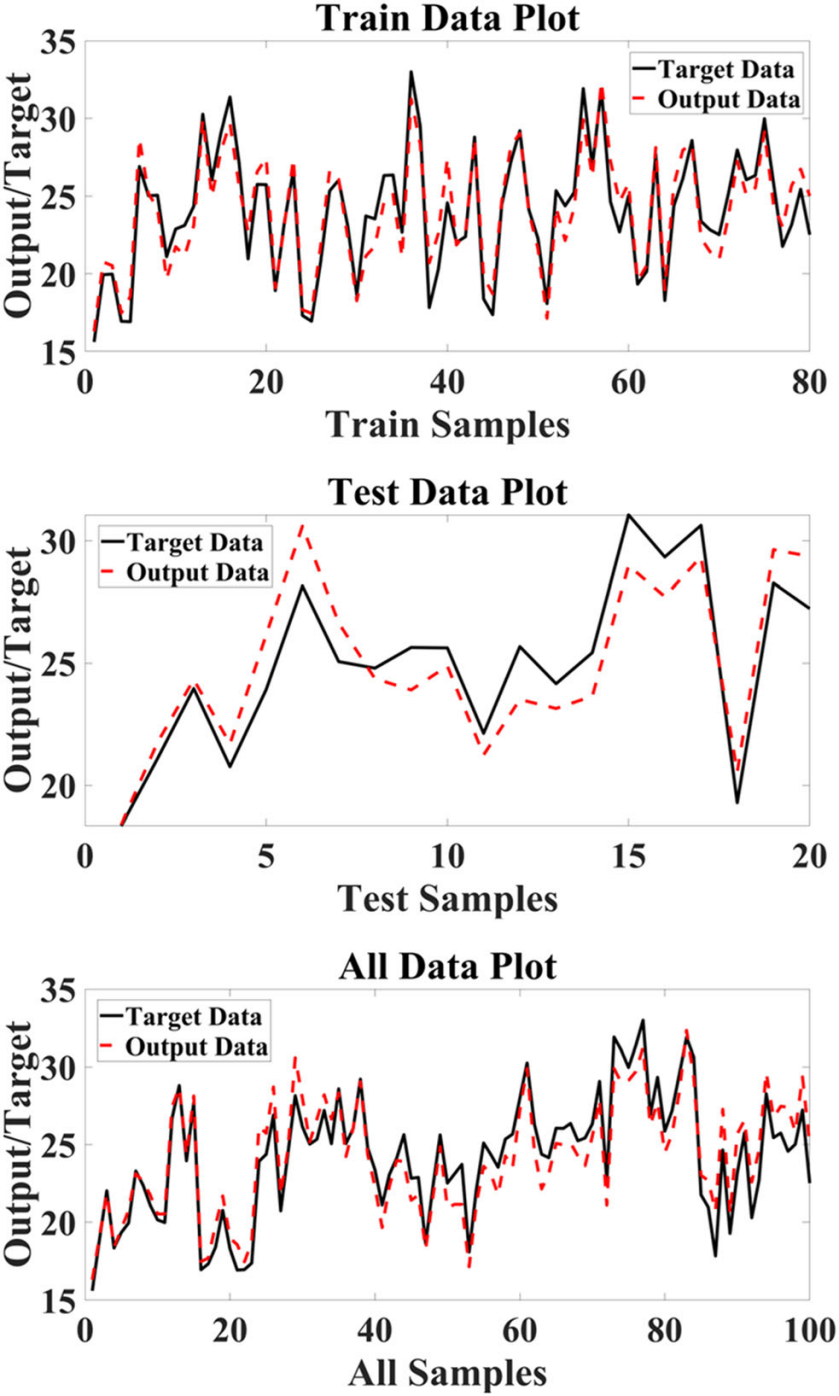
The target hyperforin content and the outputs of radial basis function (RBF) in data sets

Comparison of the real (target) and simulated (output) values of RBF in the datasets is shown in Figure 5.

### 3.3 SVM performance

The kernel function, defined in SVM structure, classifies the data into a matrix of multidimensional space. The *C* parameter, epsilon (*ε*), and parameter gamma (*γ*) are key factors that influence SVM regression performance. We employed the value of the parameter *ε* to specify the number of support vectors as described by Laref et al. (2018). SVM regression with the Gaussian function includes *γ* parameter to define the width of bell‐shaped curves. In this study, the linear function resulted in a more accurate prediction so the Gaussian function and *γ* parameter were later eliminated. In the SVM model, we looked for simple curves where the value of the parameter *C* helps to achieve these curves. To acquire hyperforin content, *C* and *ε* factors in SVM regression are defined, and the most important SVM factors are described in Table 3.

**TABLE 3:** The SVM structure results (*ε* and *C*) in training and test data sets

*ε*	*C*	Test set				Training data			
		*R* ^2^	MSE	RMSE	MAE	*R* ^2^	MSE	RMSE	MAE
0.004	922.6	.74	4.46	2.11	1.71	.76	3.43	1.85	1.29

Abbreviation: SVM, support vector machine.

**FIGURE 6:**
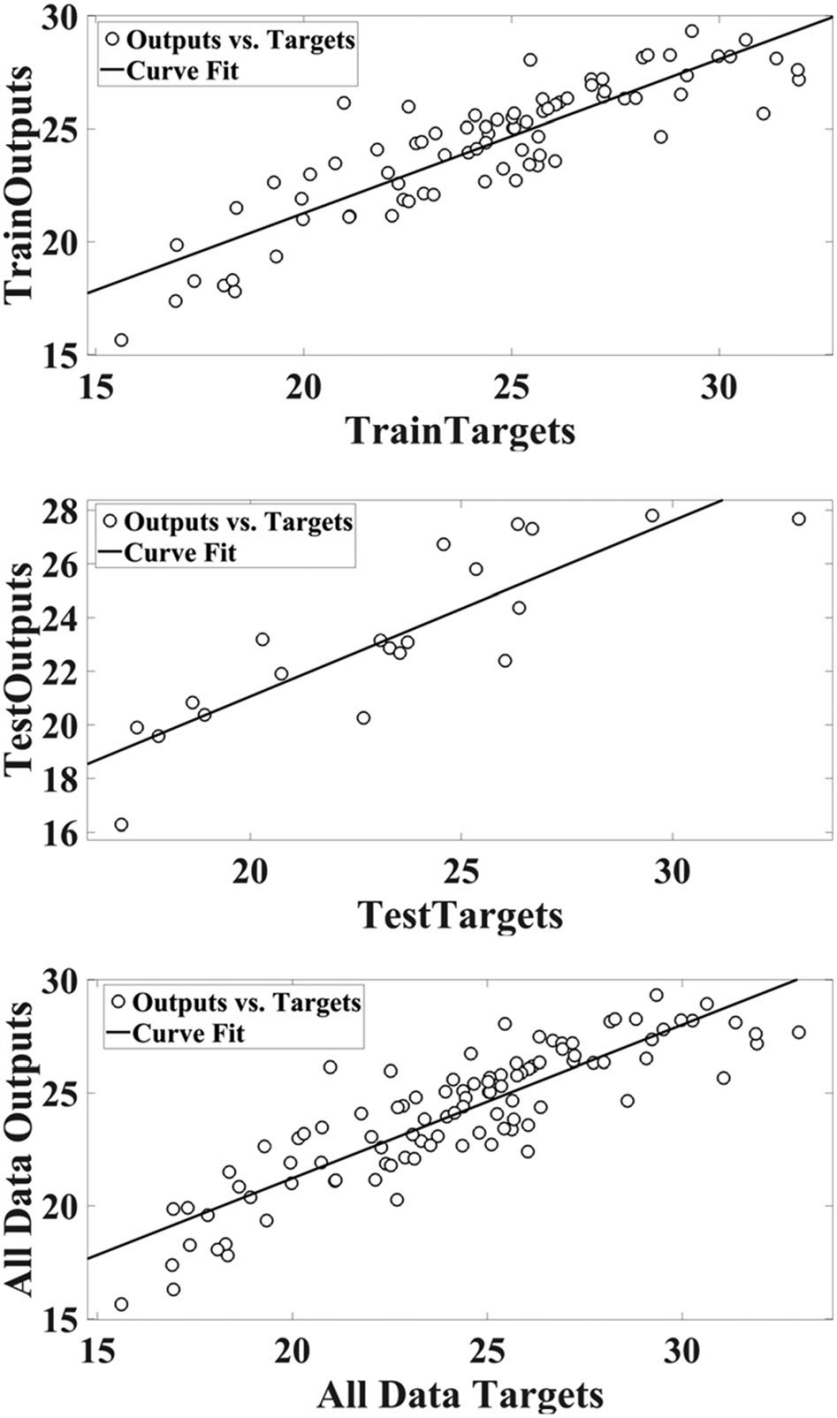
Scatter plots of targets values of hyperforin content via support vector machine (SVM) outputs

Based on the values of *R* ^2^ in training and test datasets, the best *ε* value was 0.004, and *C* value was 922.6. The other models having various *ε* and *C* displayed over‐fitting and under‐fitting in models. The scatter plot of SVM outputs via target values of the hyperforin for training, test, and total data are shown in Figure 6. The values of coefficient (*R* ^2^) suggest the correlation between the SVM outputs and target values.

**FIGURE 7:**
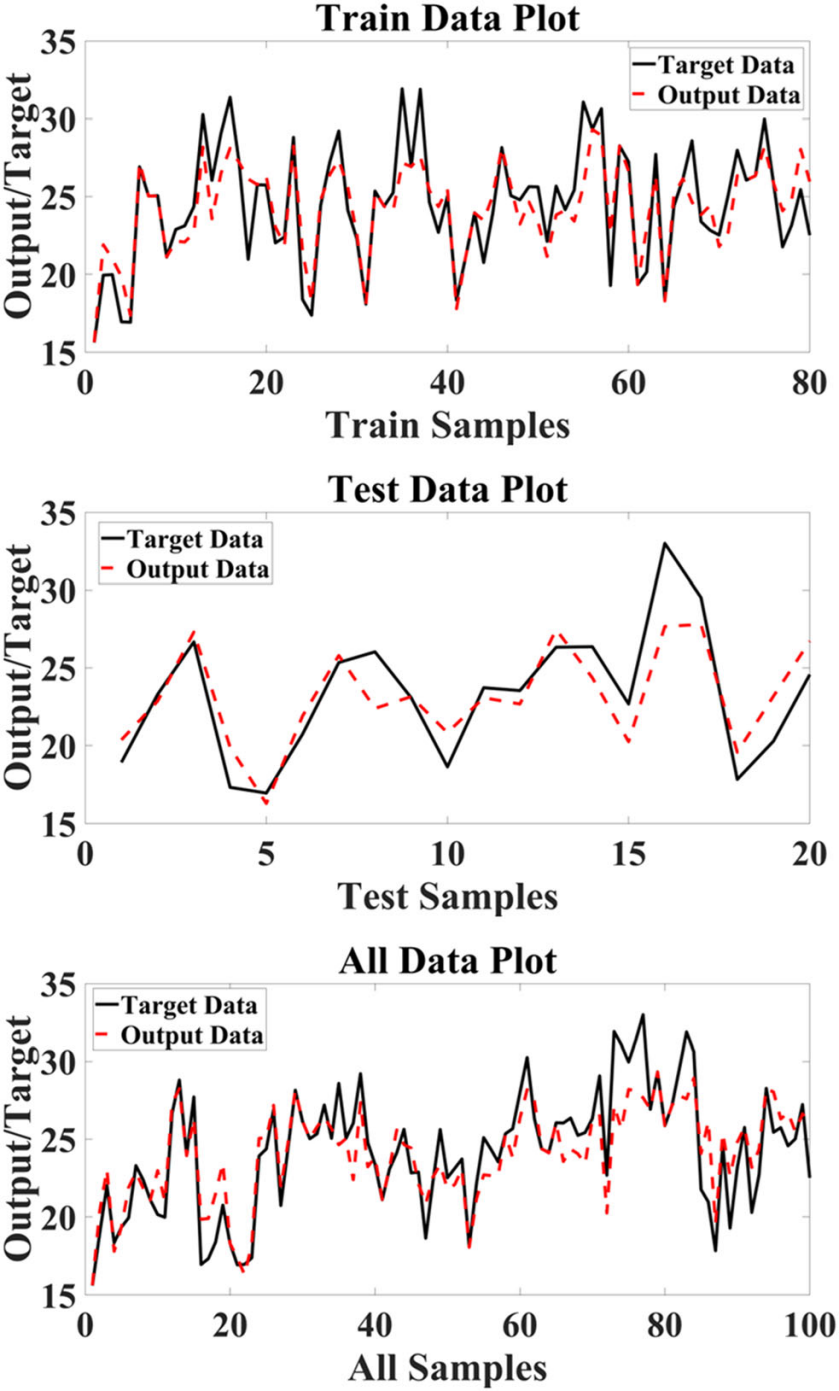
The target hyperforin content and the outputs of support vector machine (SVM) in data sets

Comparison between the simulated (output) values and the real target of SVM in datasets is shown in Figure 7.

### 3.4 Model selection

**FIGURE 8:**
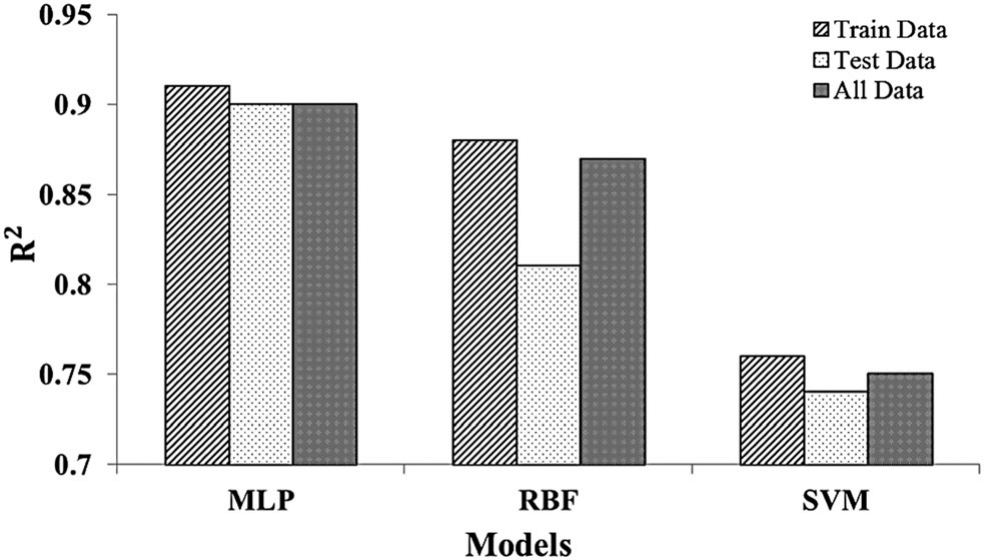
The performance measures in data sets of three artificial intelligence models

As we compared the output of the MLP, RBF, and SVM models in Figure 8, it is clearly demonstrated that the MLP model is the most suitable model measured by the highest value of *R* ^2^ in training, test, and all datasets.

### 3.5 Sensitivity analysis of MLP

**FIGURE 9:**
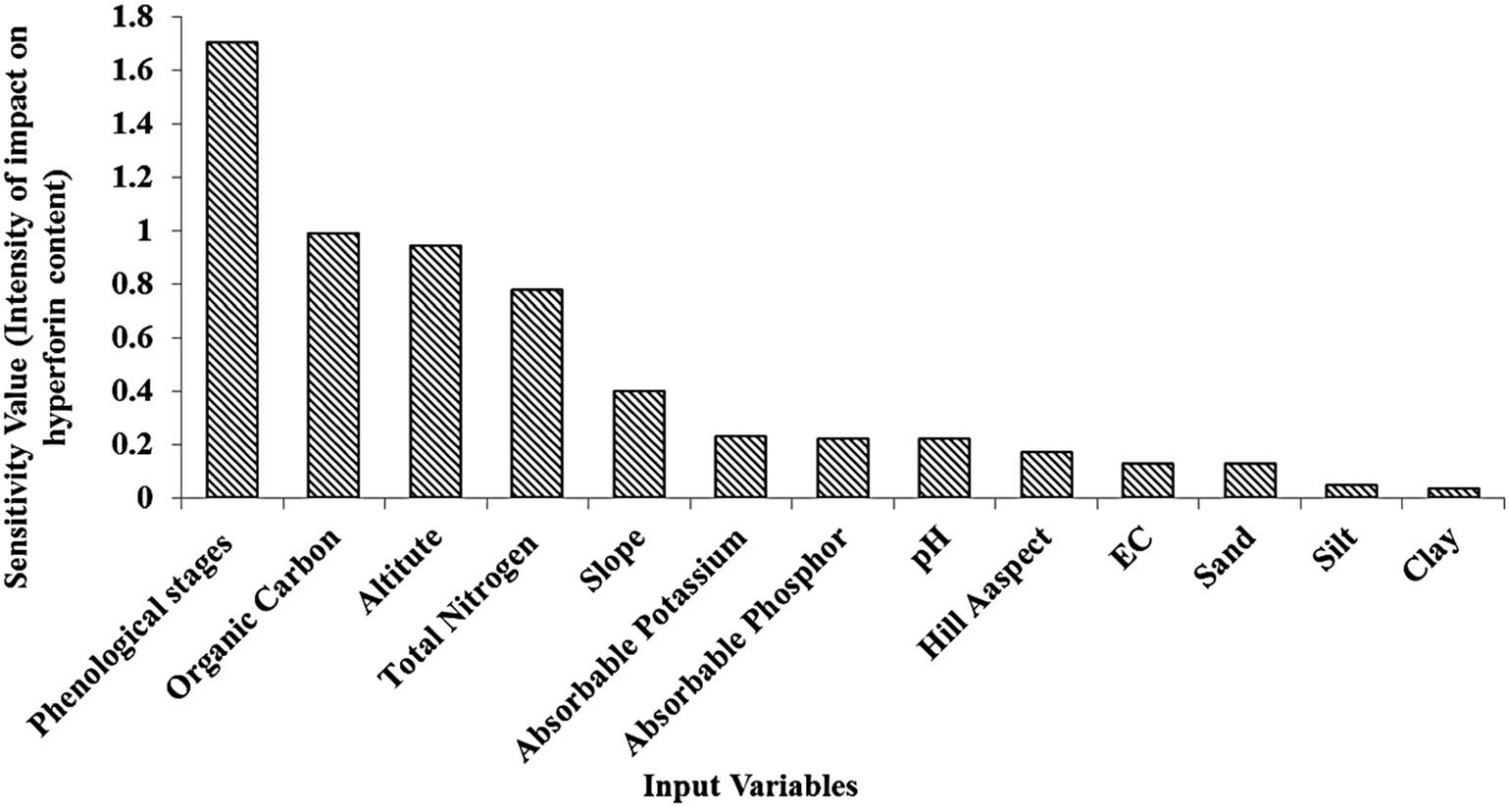
Sensitivity analysis of hyperforin content prediction (multilayer perceptron [MLP]) model

The MLP model sensitivities for input variables are displayed in Figure 9. The standard deviations of MLP outputs for the content of hyperforin are shown in Figure 9. The most critical inputs that have an impact on MLP output are phenological stages, organic carbon, altitude, and total nitrogen, respectively.

**FIGURE 10:**
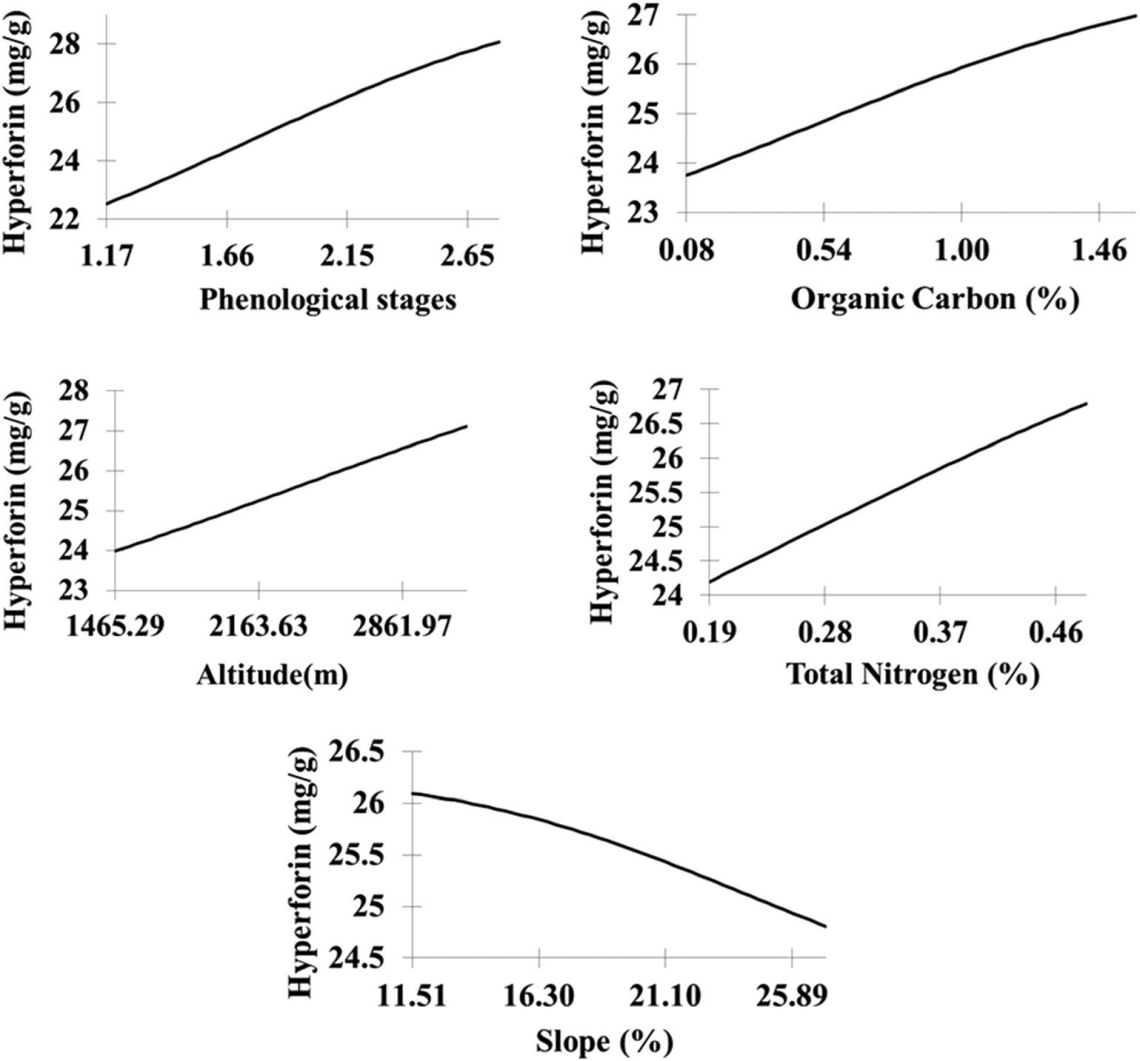
The trend of MLP output changes (hyperforin content) in response to the input variables

Figure 10 displays that the amount of hyperforin in *H. perforatum* increases by increasing phenological stages, organic carbon, altitude, and total nitrogen, but the opposite trend was observed for the slope variable.

To determine the content of hyperforin in *H. perforatum* in other areas, a GUI was designed to assist robust prediction of the hyperforin content in varied habitat conditions. The EDSS tool will be operated by pressing the “hyperforin content prediction” function in the GUI tool.

## 4 DISCUSSION

Several factors drive the spatiotemporal distribution of plant's chemical footprints, and it is almost impossible to single out a particular factor solely responsible of observed changes in composition of metabolites (Radušienė et al., 2012). The current study evaluated the effect of phenological stages, landform characteristics, and soil features on hyperforin content in *H. perforatum*. Our findings suggest that hyperforin content was significantly dependent on phenological stages, organic carbon, altitude, and total N. Out results are in agreement to another study by Ruzalepp et al., 2017 comparing chemical composition of secondary metabolites was impacted in *H. perforatum* and *Hypericum maculatum* by various phenological stages, organic carbon levels, and altitude. Results of our study are important to understand biosynthesis of secondary metabolites with higher medicinal value including hyperforin under a range of ecological habitats.

To the best of our knowledge, this study is the first report to predict hyperforin content in *H. perforatum* using artificial intelligence techniques. Recently, the use of the artificial intelligence techniques remained a subject of active research (Cline et al., 2018; Jahani et al., 2011; Rawlins et al., 2012). This technique not only is applicable in predicting active compounds in *H. perforatum* but also could be useful for other plants with various environmental conditions. We compared three models (MLP, SVM, and RBF) and revealed that the MLP as an EDSS tool outperformed in predicting hyperforin content more accurately (*R* ^2^ = .9) compared with RBF (*R* ^2^ = .81) and SVM (*R* ^2^ = .74) under plant growth, landform conditions, and soil features considered in this study. Because identification and quantification of hyperforin are expensive and require tandem mass spectrometry and skillsets, the ANN modeling could potentially serve as an alternative approach (Eftekhari et al., 2018). ANN is a mathematical approach to obtain the closest result to the expected value (Suárez et al., 2015). These models have been recently compared in another study looking at aesthetic quality prediction and vegetation density (Jahani & Saffariha, 2020a). In addition, Savić et al. (2013) reported that MLP, along with central composite design (CCD), was the most suitable model in predicting the total flavonoid extraction from green tea. Moreover, the ANN modeling approach has several advantages over regression modeling as discussed in detail previously (Jamshidi et al., 2016).

We demonstrated that the most influential factors impacting hyperforin content were phenological stages, organic carbon, altitude, and total N as measured by sensitivity analysis. This observation is in agreement with several other reports studying influence of altitude, growth stages, and genotype on hyperforin content (Büter & Büter, 2008; Filippini et al., 2010; Xenophontos et al., 2008; Zobayed et al., 2006). Similarly, other factors including nitrogen availability, phenologic stage, drought stress, altitude, and soil feature are also reported to impact hyperforin content (Cirak & Radusiene, 2019; Murch et al., 2003). We observed a positive correlation between organic carbon, altitude, total N, and the hyperforin content. Bozin et al. (2013) and Gioti et al. (2009) reported the highest concentration of hyperforin at floral budding stage, which was later confirmed by Kladar et al. (2017) reporting the highest level of active ingredients in *H. perforatum* between floral budding and flowering stage. However, other reports provided evidence of highest concentration at ripening stage (Cirak et al., 2008) and fruit development (Filippini et al., 2010). This discrimination in the literature could be explained by instability and sensitivity of hyperforin to light (Zidorn, 2010). Yesaghi (2006) studied three habitats in Iran and determined that carbon and N‐rich soil provided more suitable conditions for the vegetative growth of *H. perforatum*. Kuo et al. (2020) and Mir et al. (2019) reported a positive correlation between hyperforin content and soil organic carbon and N. Furthermore, the increasing altitude from 1400 to 2800 m led to an increase in hyperforin content from 24 to 27 mg/g, respectively, which is inconsistent with the results of Walker et al. (2001), Kleemann et al. (2014) and Xenophontos et al. (2008) who understood there wasn't any significant difference in amount of hyperforin and hypericin in different altitudes. Altitude is well known factor that can significantly impact secondary metabolism and cause variation in quality and quantity of major essential oil components of *H. perforatum* (Seyis et al., 2020). Increasing the level of secondary metabolites in plants is the adaptive response of plants to reduce temperature and increase UV‐B radiation (Zidorn, 2010). Camas et al. (2014) confirmed that chemical contents including hypericin were elevated with increasing altitude from 400 to 1250 m in *Hypericum orientale* L. and was likely due to elevated UV‐B irradiation (Yang et al., 2011).

Recently, there has been great emphasis on therapeutic properties of secondary metabolites and their variations under either genetic factor or ecological factors (Demasi et al., 2018). Mathematical tools based on artificial intelligence are useful in predicting contents of a bioactive ingredient under various ecological conditions (Odabas et al., 2009). Results of our provided a clear evidence that the MLP model could predict the hyperforin content in *H. perforatum* more accurately and can greatly reduce the cost and time require for analytical procedures. Moreover, there is a designed GUI make the MLP model user friendly. Our results could be useful for pharmacognosists, manufacturers, rangeland managers, and other fields of studies.

## 5 CONCLUSION

The recent emphasis on plant secondary metabolite and their use in pharmaceutical, medicinal and food industry require study of ecological factors for maximum yield of the active ingredients. It also requires complementary approaches for accurate prediction of bioactive constituents. Our study compared prediction of hyperforin content with the help of three models and profoundly suggests that the MLP was the most accurate model in predicting hyperforin content defined by MATLAB 2018 software. Furthermore, we observed a positive correlation between phenological stages, organic carbon, altitude, and total N with hyperforin content. Developing such approaches can greatly reduce the cost and resources required for traditional analytical platforms, and various industries such as pharmaceutical and agrochemical can potentially benefit for these models.

## CONFLICT OF INTEREST

The authors declare that they have no known competing financial interests or personal relationships that could have appeared to influence the work reported in this paper.

## AUTHOR CONTRIBUTIONS

*Data gathering, Data analysis, Article writing*: M. S.; *Data analyzing, Method curation, Article editing*: A. J.; *Lab analysis, Visualization (if applicable)*: R. J.

## Data Availability

Data will be available by authors in reasonable request.
